# Supplementary material for: A clustering-independent method for finding differentially expressed genes in single-cell transcriptome data
Source: Nat Commun. 2020 Aug 28;11:4318. doi: 10.1038/s41467-020-17900-3 (PMC7455704; doi:10.1038/s41467-020-17900-3)
Supplement: Supplementary file 2 — Supplementary Information [file 41467_2020_17900_MOESM2_ESM.pdf]

Supplementary Information for

A clustering-independent method for finding differentially expressed genes in single-cell transcriptome data

Alexis Vandenbon[1,2,]* and Diego Diez[3]

[1] Institute for Frontier Life and Medical Sciences, Kyoto University, 53 Shougoin Kawara-cho, Sakyou-ku, Kyoto 606-8507, Japan

[2] Institute for Liberal Arts and Sciences, Kyoto University, Yoshidanihonmatsu-cho, Sakyo-ku, Kyoto 606-8501, Japan

[3] Immunology Frontier Research Center, Osaka University, 3-1 Yamada-oka, Suita, Osaka 565-0871, Japan

* To whom correspondence should be addressed.

Contact: alexisvdb@infront.kyoto-u.ac.jp

Table of Contents

Supplementary Methods 3

singleCellHaystack methodology 3

Step 0: Optional standardization of the input space 3

Step 1: Setting parameters 3

Step 2: Estimating reference distribution Q 3

Step 3: Estimating distributions $P(G = T)$ and $P(G = F)$ 4

Step 4: Estimating the Kullback-Leibler divergence of gene G, $DKL(G)$ 4

Step 5: Estimating the significance of $DKL(G)$ 4

The singleCellHaystack advanced mode 5

The `haystack_2D` function for 2-dimensional input 5

Step 1: Setting parameters 5

Step 2: Estimating reference distribution Q 6

Step 3: Estimating distributions $P(G = T)$ and $P(G = F)$ 6

Step 4: Estimating the Kullback-Leibler divergence of gene G, $DKL(G)$ 7

Step 5: Estimating the significance of $DKL(G)$ 7

scRNA-seq datasets and processing 7

Step 1: Data sources 7

Step 2: Filtering of cells and genes 7

Step 3: Principal Component Analysis, t-SNE and UMAP 7

Step 4: singleCellHaystack analysis 8

Step 5: Visualization of results 8

Supplementary Notes 8

Dependency of singleCellHaystack on input space 8

Dependency of singleCellHaystack on bandwidth h 8

Dependency of singleCellHaystack on the number of grid points 9

Dependency of singleCellHaystack on the coordinates of grid points 9

Dependency of cluster-based approaches on the number of clusters 9

Supplementary Tables 10

Supplementary Figures 13

Supplementary References 26

Supplementary Methods

singleCellHaystack methodology

The two main functions in singleCellHaystack are `haystack_2D` and `haystack_highD`, for 2D and multidimensional (≥2D) input spaces, respectively. The input parameters are 1) the coordinates of cells in a multidimensional (≥2D) space (e.g. t-SNE, UMAP, PC coordinates, spatial coordinates, etc.), and 2) data indicating for each gene in which cells it was detected and not detected. We refer to Supplementary Figure 1 for an overview of the workflow. Here, we first focus on `haystack_highD`. A description of `haystack_2D` follows.

Step 0: Optional standardization of the input space

In this optional step, each dimension of the input coordinates is rescaled to mean 0 and standard deviation 1.

Step 1: Setting parameters

Steps 2-4 depend on using grid points for estimating the local density of cells using a Gaussian kernel, in function of their distance to the grid points. In the first step, `haystack_highD` decides the grid points and bandwidths for doing these calculations.

By default, 100 grid points (option grid.points) are decided in a way that results in grid points being roughly uniformly spread over the subspace in which the cells are located. In other words, grid points should not be located close to each other, but should be proximal to the cells, and no cells should be distal from all grid points. In `haystack_highD`, the default way of deciding grid points is by running k-means clustering of the input cell coordinates, and then use the resulting 100 centroids as grid points. Note that the clustering of cells itself is not important. Another approach is to use seeding, as used in k-means++ clustering, in which initial centers are picked iteratively so that they are distal from other centers [1].

A bandwidth h is decided as follows: for each cell, the distance to the closest grid point is calculated, and h is defined as the median of those distances. Normalized distances between cells and grid points are subsequently defined as the Euclidean distances divided by the bandwidth h. The density contribution of each cell to each grid point is calculated as:

$$d_{cell,i} = e^{\left(-\frac{{Dist_{cell,i}}^2}{2}\right)} \quad (1)$$

where $Dist_{cell,i}$ is the normalized distance between $cell$ and grid point i.

Step 2: Estimating reference distribution Q

The density contributions of all cells in the multidimensional space are used to estimate a reference distribution, Q. The density of cells at each grid point i in the space is calculated as: (2)

$$Q(i) = \sum_{cell} d_{cell,i}$$

After this, Q is normalized to sum to unity.

Step 3: Estimating distributions $P(G = T)$ and $P(G = F)$

The distribution of the cells in which gene G is detected, $P(G = T)$, and those in which gene G is not detected, $P(G = F)$, is estimated using the same bandwidth and grid points, as follows:

$$P(G = T, i) = \sum_{cell(G=T)} d_{cell,i} \quad (3)$$

and

$$P(G = F, i) = \sum_{cell(G=F)} d_{cell,i} \quad (4)$$

where $cell(G = T)$ and $cell(G = F)$ represent the subsets of cells in which G is detected and not detected, respectively. Subsequently $P(G = T)$ and $P(G = F)$ are normalized to sum to unity.

Step 4: Estimating the Kullback-Leibler divergence of gene G, $D_{KL}(G)$

The divergence of the expression pattern of gene G, $D_{KL}(G)$, is calculated as follows:

$$D_{KL}(G) = \sum_{s \in \{T,F\}} \sum_{i} P(G = s, i) \log\left(\frac{P(G=s,i)}{Q(i)}\right) \quad (5)$$

If the cells in which G is detected (and not detected) do not show a biased distribution, and approximately follow the reference distribution Q, then $D_{KL}(G)$ is close to 0. As the discrepancy to the reference distribution Q increases, the value of $D_{KL}(G)$ also increases.

Step 5: Estimating the significance of $D_{KL}(G)$

Finally, `haystack_highD` evaluates the statistical significance of the $D_{KL}(G)$ values. We cannot naively regard high $D_{KL}(G)$ values as significant, because there is a tendency for genes expressed in very few cells, and for genes expressed in almost all cells, to have a high $D_{KL}(G)$.

Instead, `haystack_highD` evaluates the significance of observed $D_{KL}(G)$ values by comparing them to randomized data. First, let c_G represent the number of cells in which each gene G is detected in the scRNA-seq data. In practice, 50 sets of randomized genes are made that are expressed in c cells; c being 200 values taken from the actual c_G values of the data, evenly spread across the range of c_G values. The $D_{KL}(G)$ of these randomized genes are recorded. If there are less than 200 unique c_G values in the data, then all are used.

For each c value, the randomized $log_2(D_{KL}(G))$ values follow an approximately normal distribution. We can therefore use the mean and standard deviation of randomized $log_2(D_{KL}(G))$ values to estimate a p-value of the actually observed $D_{KL}(G)$ values. The mean and standard deviation of randomized $log_2(D_{KL}(G))$ values are modeled as a function of c using B-splines (using the `splines` R package). By default, 10 degrees of freedom are used for the splines, although this is lowered if there are few c values.

Using the B-splines, expected means and standard deviations are predicted for each gene, in function of its c_G. P-values are then calculated using the `pnorm` function in R.

The singleCellHaystack advanced mode

In practice, in some cells more genes are detected than in others (for examples, see Figure 3A, and Figure 6, left). The default reference distribution Q does not take this into account, which can lead to genes being judged to be DEGs, even if they are merely detected in cells that have many detected genes.

`haystack_highD` can take this into account by weighting the density contributions of cells by the number of genes detected in each cell:

$$d_{cell,i}^{advanced} = e^{\left(-\frac{Dist_{cell,i}^{2}}{2}\right)} \times g_{cell} \quad (6)$$

where g_{cell} is the number of genes detected in $cell$. This assigns a larger influence to cells with many detected genes in the calculation of Q. Estimation of $P(G = T)$ and $P(G = F)$ is done as in the default mode.

A second difference is in the construction of the randomized genes (see Step 5 above). In the default mode randomized genes are simulated by randomly picking c cells from all cells in the input data according to a uniform probability (i.e. each cell has the same chance of being selected). In the advanced mode, the probabilities reflect the number of genes detected in each cell (i.e. cells in which many genes are detected have a higher chance of being selected). As a result, randomized genes tend to follow the detection levels of the actual cells more closely.

The advanced mode can be activated by giving the vector of g_{cell} values as input to the use.advanced.sampling parameter of the `haystack_highD` and `haystack_2D` functions.

The `haystack_2D` function for 2-dimensional input

In addition to `haystack_highD`, which is aimed at >2D input, singleCellHaystack includes the function `haystack_2D`, which was designed for 2D input coordinates. The general concept is the same. Below, we discuss differences in steps 1-4. Step 5 is the same as introduced above.

Step 1: Setting parameters

For `haystack_2D`, steps 2-4 depend on the mapping of 2D coordinates onto a grid, and estimating the local density of cells using a Gaussian kernel, in function of their

distance to the grid points. In the first step, `haystack_2D` decides the parameters for doing these calculations. Bandwidths h_x and h_y for the X and Y axes are decided using the rule-of-thumb of the `bandwidth.nrd` function (MASS package in R). The number of grid points is decided so that there are 25 grid points between the 10% and 90% percentile of coordinates along both axes. The grid is then further extended until it covers all points. The goal of this strategy is to reduce the influence of outliers on the definition of the grid.

The density contributions of each cell to each grid point along both axes is calculated as:

$d_{cell,i} = e^{\left(-\frac{\left(\frac{x_{cell}-x_i}{h_x}\right)^2}{2}\right)}$ for the X-axis grid points, and (7)

$d_{cell,j} = e^{\left(-\frac{\left(\frac{y_{cell}-y_j}{h_y}\right)^2}{2}\right)}$ for the Y-axis grid points, where x_{cell} and y_{cell} represent the coordinates of each cell $cell$, x_i and y_j the coordinates of grid points of the X-axis and Y-axis, respectively. (8)

Step 2: Estimating reference distribution Q

The density distribution of all cells in the 2D space is used as a reference distribution, Q. The density at each grid point in the 2D space is calculated as:

$$Q(i,j) = \sum_{cell} \sum_{i,j} d_{cell,i} \times d_{cell,j} \tag{9}$$

To each $Q(i,j)$ value, a small pseudo count is added, defined as the 1% percentile value of non-zero $Q(i,j)$ values. After this, Q is normalized to sum to unity.

Step 3: Estimating distributions $P(G = T)$ and $P(G = F)$

The distributions of the cells in which gene G is detected, $P(G = T)$, and those in which gene G is not detected, $P(G = F)$, are estimated using the same bandwidths and grid points, as follows:

$$P(G = T, i, j) = \sum_{cell(G=T)} \sum_{i,j} d_{cell,i} \times d_{cell,j} \tag{10}$$

and

$$P(G = F, i, j) = \sum_{cell(G=F)} \sum_{i,j} d_{cell,i} \times d_{cell,j} \tag{11}$$

where $cell(G = T)$ and $cell(G = F)$ represent the subsets of cells in which G is detected and not detected, respectively. The same pseudo count is added as for Q, and subsequently $P(G = T)$ and $P(G = F)$ are normalized to sum to unity.

Step 4: Estimating the Kullback-Leibler divergence of gene G, $D_{KL}(G)$

The divergence of the expression pattern of gene G, $D_{KL}(G)$, is calculated as follows:

$$D_{KL}(G) = \sum_{s\in\{T,F\}} \sum_{i,j} P(G = s, i, j) \log\left(\frac{P(G=s,i,j)}{Q(i,j)}\right) \quad (12)$$

Step 5: Estimating the significance of $D_{KL}(G)$

This step is the same as for `haystack_highD` (see above).

scRNA-seq datasets and processing

Datasets were processed as described below. In many steps, we followed the recommendations described by Kobak and Berens [2].

Step 1: Data sources

- Tabula Muris data was downloaded from https://tabula-muris.ds.czbiohub.org/ [3].
- Mouse Cell Atlas data file MCA_BatchRemove_dge.zip was downloaded from https://figshare.com/articles/MCA_DGE_Data/5435866. This data has been treated to reduce batch effects [4].
- The Nestorowa *et al*. dataset (processed read counts) was downloaded from GEO, accession number GSE81682 [5].

Step 2: Filtering of cells and genes

Library sizes of each cell were calculated by summing the number of reads or UMI (Universal Molecular Identifier) counts over all genes. Counts were then converted to counts per million counts (CPM). Genes were defined to be detected in a cell if their CPM was above a threshold CPM. We used the median CPM of each gene in the dataset as threshold, except for the spatial transcriptomics analysis where we defined a gene detected if the counts where > 0.

To focus on dataset sizes that are representative of typical current single-cell datasets we randomly selected 20,000 cells in datasets with more than 20,000 cells. For the Tabula Muris microfluidic droplet data, we selected the 20,000 cells with the most detected genes.

We filtered out cells with fewer than 100 detected genes, and genes detected in fewer than 10 cells.

Step 3: Principal Component Analysis, t-SNE and UMAP

We selected 1,000 genes with large variance given their mean using dropout rates and mean CPM across non-zero counts, as described by Kobak and Berens [2]. The $\log_2$ CPM values of these 1,000 genes (adding pseudocount of 1) were used as input for PCA, without scaling. Subsequently, t-SNE and UMAP were run on the first 50 PCs. For t-SNE, we used the Rtsne package (version 0.15) using perplexity 30, for a maximum of 500 iterations [6]. For UMAP, we used the umap package (version 0.2.0.0) [7].

Step 4: singleCellHaystack analysis

singleCellHaystack was applied on the datasets using the `haystack` function, with inputs: 1) the coordinates of cells in a ≥2D space (2D t-SNE coordinates, 2D UMAP coordinates, or 5, 10, 15, 25, and 50 PCs) and 2) the detection data of each gene in each cell. This includes all genes that passed the filtering step (i.e. not only the 1,000 genes used as input for PCA). `haystack` was run both using the default mode and the advanced mode, which takes into account the general detection levels of genes (see main manuscript and explanation above). Runtimes were recorded for each run.

Step 5: Visualization of results

For the results returned by `haystack` by the default mode and the advanced mode, the following was done, separately:

- Top-scoring DEGs were selected, using the function `show_result_haystack`. For this, the p-value threshold 1e-6 was used.
- Significant DEGs were grouped into clusters by similarity of their expression pattern in the input space by hierarchical clustering. This was done using the function `hclust_haystack`. For the example application on the Mouse Cell Atlas Testis 1 dataset (Supplementary Figure 6), the function `kmeans_haystack` for k-means clustering was used. In all cases the number of clusters was arbitrarily set to 5.
- The average distribution of the genes in each of the clusters was visualized using function `plot_gene_set_haystack` (see for example Supplementary Figure 4).
- Within each cluster, the most significant DEG was plotted using `plot_gene_haystack` (see for example Figure 3 and Supplementary Figures 5 and 6).

Supplementary Notes

Dependency of singleCellHaystack on input space

As expected, different input spaces result in different results (Supplementary Figure 7A). Similar differences were observed for cluster-based DEG prediction approaches when they were given clusters of cells obtained from different input spaces (e.g. clustering done on the first 5 PCs, first 10 PCs, etc.). Generally, similar input spaces (t-SNE vs UMAP, or 5 PCs vs 10 PCs) returned more consistent results, while different input spaces (5 PCs vs 50 PCs) resulted in more discrepancies. Although generally there was a correlation in the p-values obtained from different input spaces, there was a notable deviation seen especially in the 50 PC input for the Testis 1 dataset. The 11th to 50th PC of this dataset contains variation which is obviously not included in the first 5 or 10 PCs and which is not completely captured by t-SNE and UMAP.

Dependency of singleCellHaystack on bandwidth h

The estimation of the gene expression distributions depends on a bandwidth h (see Supplementary Methods). Our method uses a heuristic to pick a suitable (neither too

large nor too small) bandwidth value that is in the order of magnitude of the typical distance between cells and the nearest grid point. Supplementary Figure 7B shows how results change when the bandwidth is increased or decreased. In general, the default bandwidth setting returns consistent results with larger or smaller bandwidths (i.e. top scoring genes are the same). However, when the bandwidth is very narrow more discrepancies appear. With very narrow bandwidths, distances between cells and grid points increase to a point where some cells have no proximal grid points, resulting in some expression patterns being less well captured.

Dependency of singleCellHaystack on the number of grid points

For high-dimensional input spaces (ex: 50 PCs) it is impossible to use an extensive grid that covers the entire input space. Instead, singleCellHaystack uses a limited set of grid points (see Supplementary Methods) covering the parts of the input space that contain cells. By default, singleCellHaystack uses 100 such grid points, but different values can be specified by the user. Supplementary Figure 7C compares results when using 25, 50, 100, 150, and 200 grid point. In general, top scoring DEGs are consistent regardless of the number of grid points, but some discrepancies can be seen especially between runs using 25 vs 200 grid point. Using too few grid points could result in smaller groups of cells being not well covered. For very large and heterogeneous dataset, increasing the number of grid points is advised. On the other hand, using too many grid points could result in longer runtimes.

Dependency of singleCellHaystack on the coordinates of grid points

The grid points used by `haystack_highD` are not deterministic (see Supplementary Methods). Unless a seed value is set for R's random number generator, each run will use different grid point coordinates and will return different results. To evaluate the dependency of results on grid point coordinates, we ran our method using 5 different seed values (Supplementary Figure 7D). We observed that results were stable.

Dependency of cluster-based approaches on the number of clusters

For comparison, we evaluated how a typical cluster-based method (Seurat's `FindAllMarkers` function using the default Wilcoxon Rank Sum test) depends on the number of predicted clusters. For each dataset, we applied Seurat's `FindAllMarkers` function on the default number of predicted clusters, as well as on the default reduced by 2 and 1, or increased by 1 and 2 (Supplementary Figure 7E). For each dataset, we found that increasing or decreasing the number of clusters resulted in considerable differences in the top-scoring DEGs.

Note that the number of clusters returned by Seurat's `FindAllMarkers` function is not deterministic, and the number of clusters and cell-to-cluster assignments change when different random number seed values are used. The correct number of clusters in a real single-cell dataset is therefore hard to determine, illustrating the importance of this comparison: different clustering results can result in considerably different DEGs being predicted.

Supplementary Tables

	Name of method	Short description	Implementation (version)	Comment	Ref.
1	singleCellHaystack		R package singleCellHaystack (0.3.0)		this paper
2	DEsingle	Zero-inflated negative binomial model	R package DEsingle (1.4.0)		[8]
3	EMDomics	Non-parametric Earth Mover's Distance	R package EMDomics (2.14.0)		[9]
4	scDD	Dirichlet Process mixture model	R package scDD (1.8.0)		[10]
5	edgeR LRT	Empirical Bayes likelihood ratio test	R package edgeR (3.26.8)	Designed for bulk samples	[11]
6	edgeR QLF	Empirical Bayes quasi-likelihood F-test	R package edgeR (3.26.8)	Designed for bulk samples	[11]
7	Monocle 2	Generalized linear model	R package monocle (2.12.0)	Function differentialGeneTest	[12]
8	MAST	Hurdle model	R packages MAST (1.10.0) and Seurat (3.1.4)	Using Seurat's function FindAllMarkers option "MAST"	[13]
9	wilcox	Wilcoxon Rank Sum test	R package Seurat (3.1.4)	Using Seurat's function FindAllMarkers option "wilcox"	[14]
10	bimod	Likelihood-ratio test	R package Seurat (3.1.4)	Using Seurat's function FindAllMarkers option "bimod"	[14,15]
11	ROC	ROC classifier	R package Seurat (3.1.4)	Using Seurat's function FindAllMarkers option "roc"	[14]
12	t	Student's t-test	R package Seurat (3.1.4)	Using Seurat's function FindAllMarkers option "t"	[14]
13	negbinom	Negative binomial generalized linear model	R package Seurat (3.1.4)	Using Seurat's function FindAllMarkers option "negbinom"	[14]
14	poisson	Poisson generalized linear model	R package Seurat (3.1.4)	Using Seurat's function FindAllMarkers option "poisson"	[14]
15	LR	Logistic regression framework	R package Seurat (3.1.4)	Using Seurat's function FindAllMarkers option "LR"	[14]

Supplementary Table 1: Overview of the DEG prediction methods employed in this study.

	Dataset size (number of cells)									
**Method (options)**	**1000**	**2000**	**3000**	**4000**	**5000**	**6000**	**7000**	**8000**	**9000**	**10000**
singleCellHaystack (default)	0.8572	0.9181	0.9169	0.9156	0.9230	0.8970	0.8977	0.8444	0.8420	0.8358
singleCellHaystack (advanced)	0.8548	0.9190	0.9155	0.9158	0.9239	0.8959	0.8965	0.8471	0.8409	0.8326
DEsingle	0.8968	0.9157	0.8944	0.8628	0.8747	0.8306	0.8710	0.7846	0.7742	0.7779
EMDomics	0.7625	0.7914	0.7637	0.7373	0.7769	0.7236	0.7354	0.6388	0.6434	0.6555
scDD	0.9063	0.9144	0.8926	0.8628	0.8654	0.8190	0.8337	0.7563	0.7399	0.7541
edgeR (LRT)	0.5540	0.6462	0.6482	0.6364	0.6640	0.6455	0.6122	0.5115	0.5261	0.5387
edgeR (QLF)	0.5465	0.6272	0.6443	0.6288	0.6745	0.6438	0.6111	0.5196	0.5364	0.5513
monocle 2	0.9039	0.9124	0.8863	0.8619	0.8674	0.8217	0.8345	0.7521	0.7393	0.7469
MAST (default filter)	0.6324	0.6672	0.6548	0.6424	0.6736	0.6481	0.6279	0.5870	0.6056	0.6163
MAST (no filter)	0.8445	0.8715	0.8479	0.8142	0.8456	0.7975	0.8274	0.7384	0.7296	0.7463
wilcox (default filter)	0.6324	0.6671	0.6548	0.6425	0.6737	0.6479	0.6276	0.5870	0.6056	0.6161
wilcox (no filter)	0.8455	0.8751	0.8584	0.8179	0.8493	0.8031	0.8307	0.7464	0.7374	0.7483
bimod (default filter)	0.6325	0.6673	0.6549	0.6426	0.6737	0.6482	0.6282	0.5871	0.6058	0.6167
bimod (no filter)	0.8521	0.8743	0.8600	0.8316	0.8491	0.8071	0.8341	0.7469	0.7442	0.7584
ROC (default filter)	0.6324	0.6673	0.6548	0.6425	0.6738	0.6474	0.6280	0.5873	0.6055	0.6158
roc (no filter)	0.8590	0.8798	0.8546	0.8070	0.8374	0.7843	0.8072	0.7023	0.7016	0.7187
t (default filter)	0.6324	0.6672	0.6548	0.6427	0.6739	0.6476	0.6277	0.5871	0.6057	0.6155
t (no filter)	0.8472	0.8739	0.8530	0.8216	0.8548	0.8069	0.8277	0.7381	0.7371	0.7492
negbinom (default filter)	0.8105	0.8140	0.8037	0.8029	0.8317	0.7865	0.7867	0.7226	0.7221	0.7242
negbinom (no filter)	0.8753	0.8926	0.8701	0.8246	0.8530	0.8043	0.8278	0.7342	0.7199	0.7374
poisson (default filter)	0.8089	0.8130	0.8026	0.8030	0.8315	0.7865	0.7886	0.7255	0.7259	0.7278
poisson (no filter)	0.8660	0.8915	0.8695	0.8312	0.8480	0.8079	0.8262	0.7449	0.7409	0.7488
LR (default filter)	0.6324	0.6671	0.6548	0.6424	0.6735	0.6479	0.6277	0.5869	0.6053	0.6158
LR (no filter)	0.8402	0.8719	0.8498	0.8143	0.8432	0.7947	0.8245	0.7340	0.7210	0.7400

Supplementary Table 2: Median AUC values of all evaluated DEG prediction methods applied on artificial dataset. Median AUC values are based on 20 datasets (n=20) for every dataset size, except for scDD on datasets of size 9,000 (18 datasets) and 10,000 (11 datasets).

	Dataset size (number of cells)									
**Method (options)**	**1000**	**2000**	**3000**	**4000**	**5000**	**6000**	**7000**	**8000**	**9000**	**10000**
singleCellHaystack (default)	33.5	60.6	83.3	106	134	177	201	210	231	283
singleCellHaystack (advanced)	35.1	70.3	102	141	189	251	299	337	400	472
DEsingle	9785	30529	55688	86718	141838	216955	301745	442996	538642	636632
EMDomics	3799	7199	10271	12106	16558	24256	35740	46313	52381	59670
scDD	6657	18739	34117	49020	78585	130142	185548	290642	351058	383494
edgeR (LRT)	271	805	1370	2223	2240	2475	2825	3991	5533	6671
edgeR (QLF)	273	758	1313	2112	2209	2488	3133	4630	6481	7743
monocle 2	402	812	1314	1654	2530	3645	4832	7417	8204	9390
MAST (default filter)	9.8	19.8	36.2	46.1	71.6	100	145	196	279	330
MAST (no filter)	496	974	1678	2128	3570	5044	6827	9678	12588	14942
wilcox (default filter)	5.0	13.1	26.6	36.8	58.9	84.4	121	175	244	291
wilcox (no filter)	839	2152	4205	5702	9553	15408	21548	32987	40745	50014
bimod (default filter)	3.1	7.8	15.1	23.0	36.7	61.7	86.9	121	176	206
bimod (no filter)	825	2146	4261	5832	9441	15800	21595	34989	41056	49543
ROC (default filter)	3.8	9.6	17.7	25.8	41.0	64.0	92.5	134	177	220
roc (no filter)	1235	3128	5969	8545	13718	21968	30579	50189	58270	72164
t (default filter)	3.1	7.9	14.5	24.1	36.3	54.8	82.2	124	167	201
t (no filter)	804	2113	3942	5718	9476	15011	21029	34687	40064	49044
negbinom (default filter)	17.4	43.4	79.3	113	166	245	354	429	642	733
negbinom (no filter)	1232	2935	5386	7928	12947	19401	27298	43740	52852	64504
poisson (default filter)	8.0	18.3	30.5	43.1	64.1	94.3	134	192	241	307
poisson (no filter)	768	1767	3272	4836	7991	12065	16980	27517	32410	41196
LR (default filter)	5.1	12.2	21.9	32.2	49.9	72.0	107	159	205	266
LR (no filter)	859	1951	3568	5297	8493	13089	18204	29775	35514	44990

Supplementary Table 3: Median runtimes (in seconds) of all evaluated DEG prediction methods applied on artificial dataset. Median runtimes are based on 20 datasets (n=20) for every dataset size, except for scDD on datasets of size 9,000 (18 datasets) and 10,000 (11 datasets).

Supplementary Figures

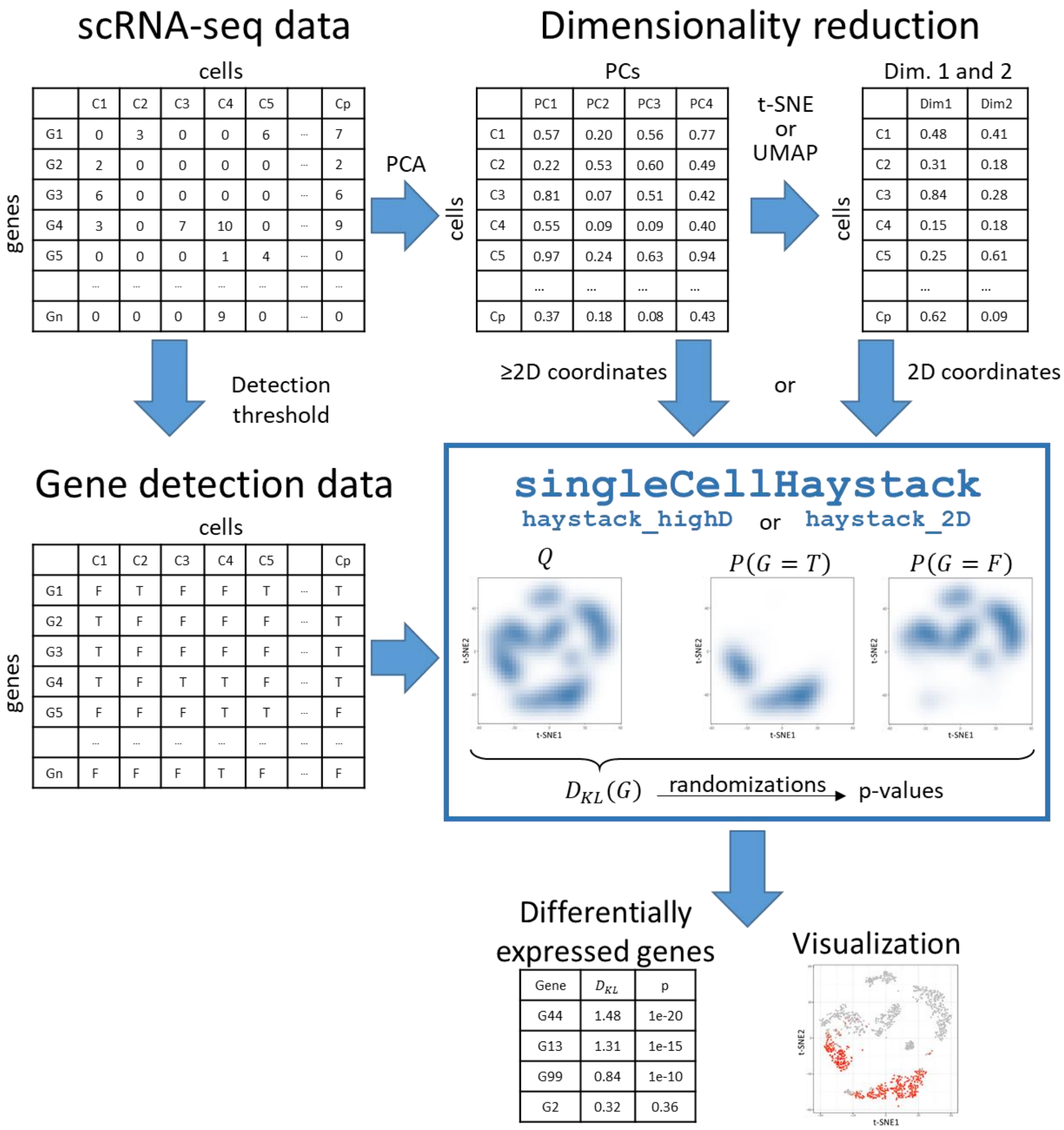

Supplementary Figure 1: Overview of the singleCellHaystack workflow. Before applying singleCellHaystack, cell coordinates in multidimensional space are obtained through PCA and (optionally) t-SNE or UMAP (or similar approaches). Read counts or UMI counts are converted to gene detection data (detected or not detected). Subsequently, the input to singleCellHaystack are the detection data, and multidimensional coordinates (`haystack_2D` for 2D coordinates and `haystack_highD` for ≥2D coordinates). The output is a list of all genes, their D_{KL} and p-value. singleCellHaystack contains additional functions for visualization and clustering of genes according to their expression pattern in the input space.

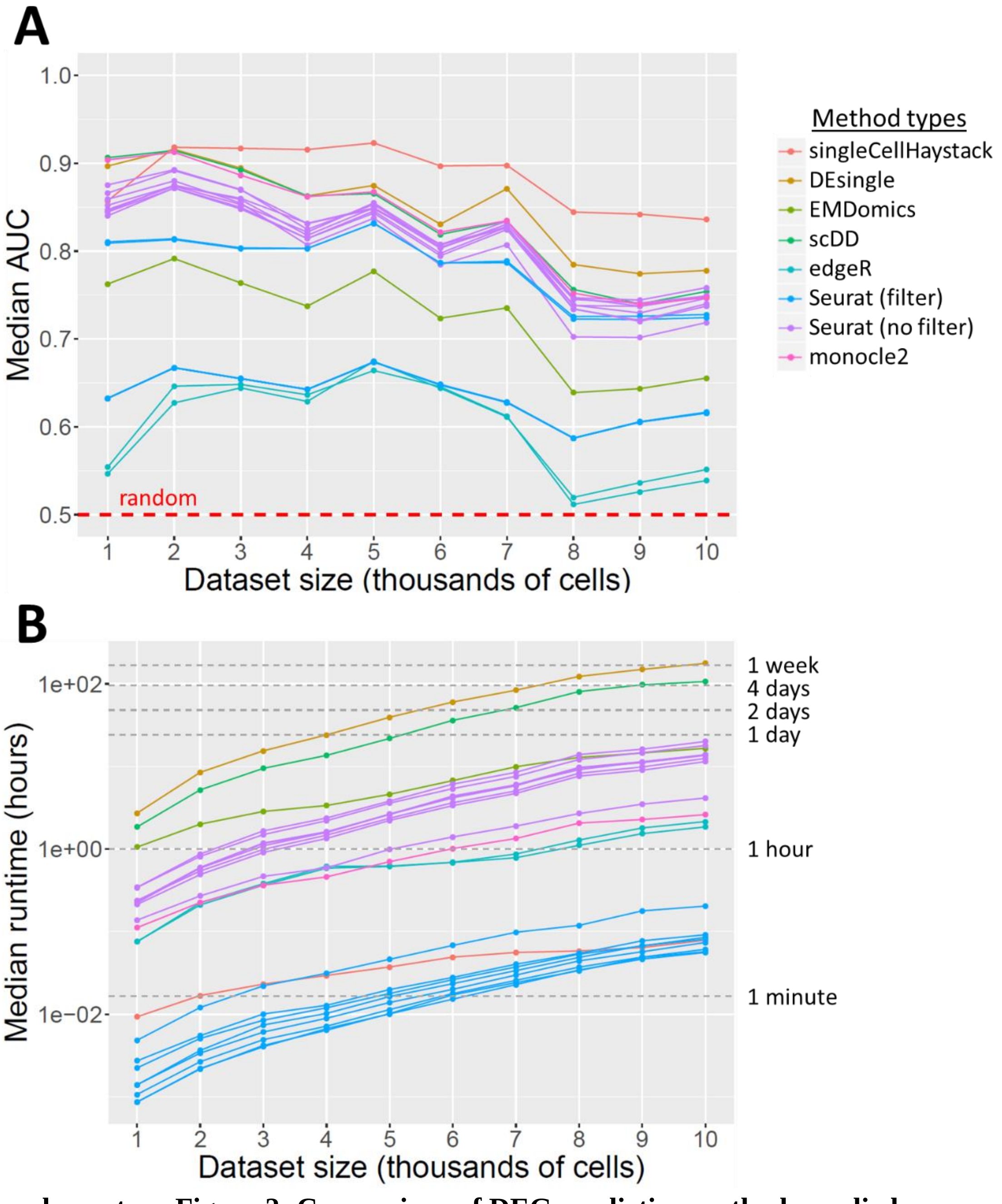

Supplementary Figure 2: Comparison of DEG prediction methods applied on artificial datasets. Similar plots are shown as in Figure 2 in the main paper. Median AUC values (A) and median runtimes (B) are shown in function of dataset size. For each dataset size, medians are based on 20 datasets (n=20), except for scDD on datasets of size 9,000 (18 datasets) and 10,000 (11 datasets). Line colors represent methods types ("edgeR" represents the LRT and QLF methods implemented in edgeR; "Seurat (filter)" represents all methods implemented in the `FindAllMarkers` function with the default filtering step; "Seurat (no filter)" represents the same methods without the default filtering step. Names of methods are not listed in the figure because behavior within types of methods was similar. For more details we refer to Tables S2 and S3.

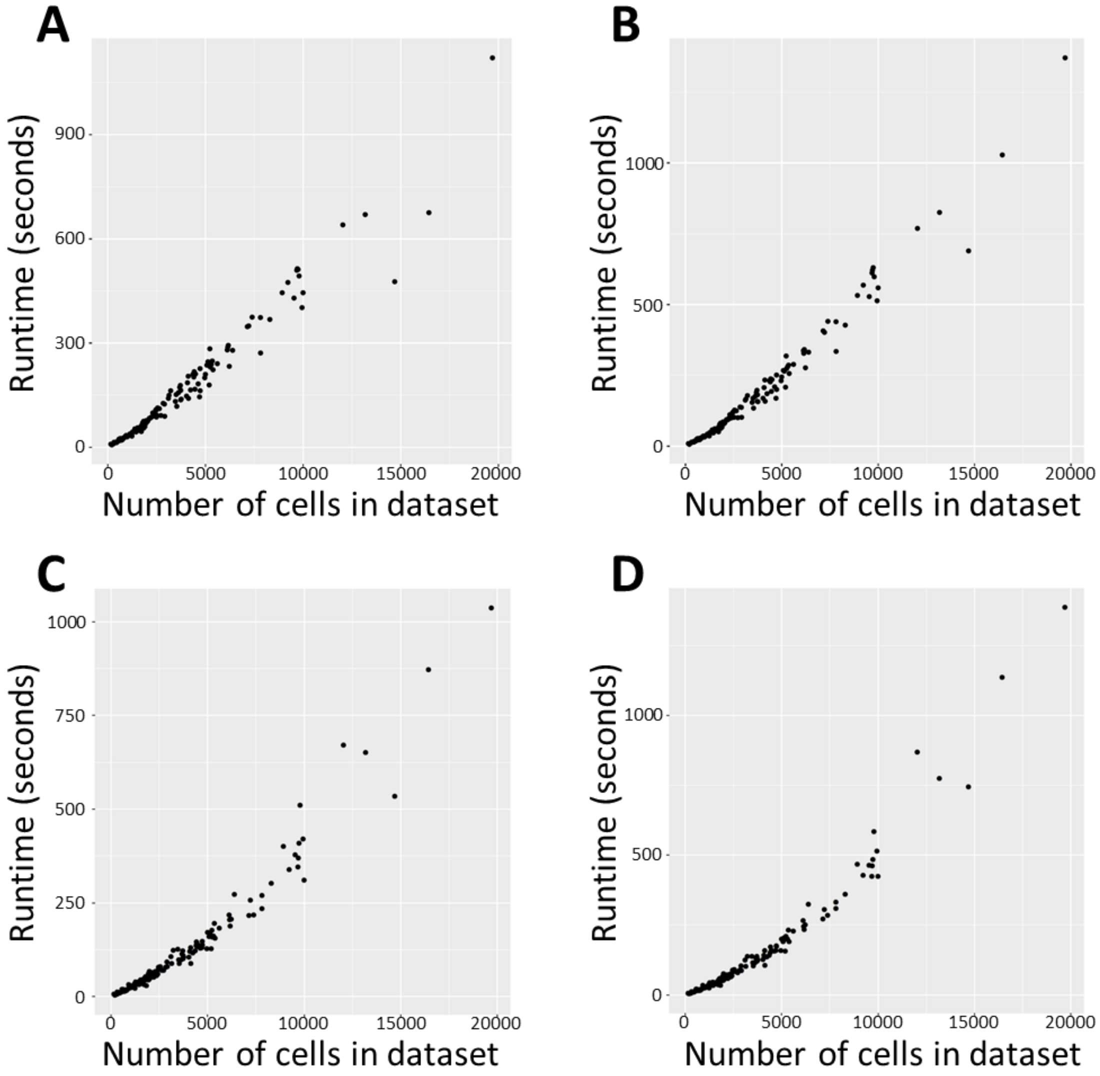

Supplementary Figure 3: Runtimes of singleCellHaystack. (A-B) Runtimes for `haystack_highD` on 50 PC input data, for the default mode (A) and the advanced mode (B) on the 136 single-cell datasets. (C-D) Runtimes for `haystack_2D` on 2D t-SNE coordinates, for the default mode (C), and the advanced mode (D) on the same 136 datasets. Runtimes were measured on a Fujitsu Esprimo WD2/M (Intel® Core™ i7-4770 CPU, 3.40GHz).

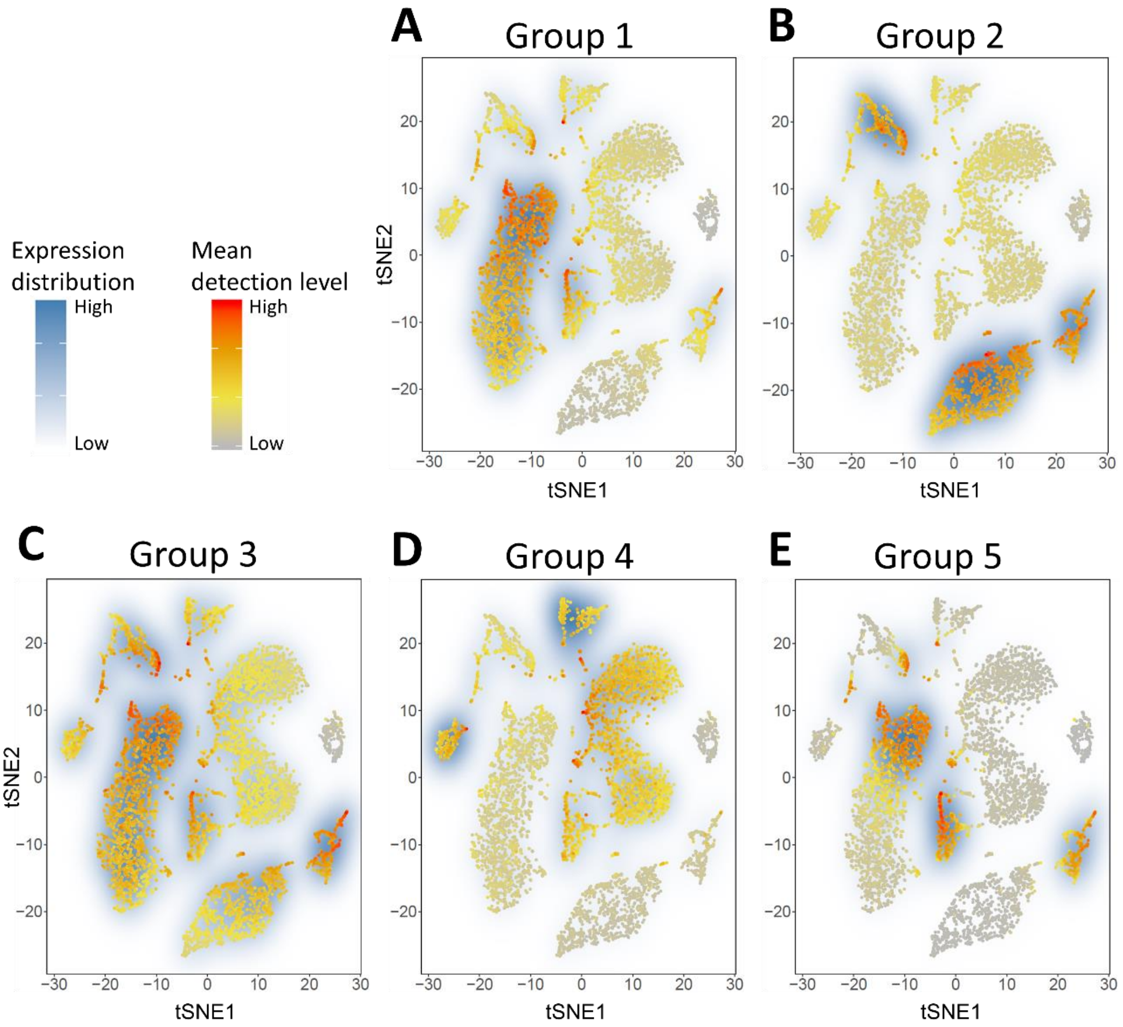

Supplementary Figure 4: Clustering of DEGs in the Tabula Muris bone marrow tissue dataset. DEGs were grouped into 5 clusters using the `hclust_haystack` function, according to their expression distribution in the input space (50 first PCs). (A-E) For each of the 5 resulting clusters, the mean detection level (the fraction of genes in the cluster detected in each cell) is shown (color scale from grey to red), as well as the averaged estimated density distribution of the genes in each cluster (color scale from white to blue). Genes shown in Figure 3 are the most significant DEGs in each cluster.

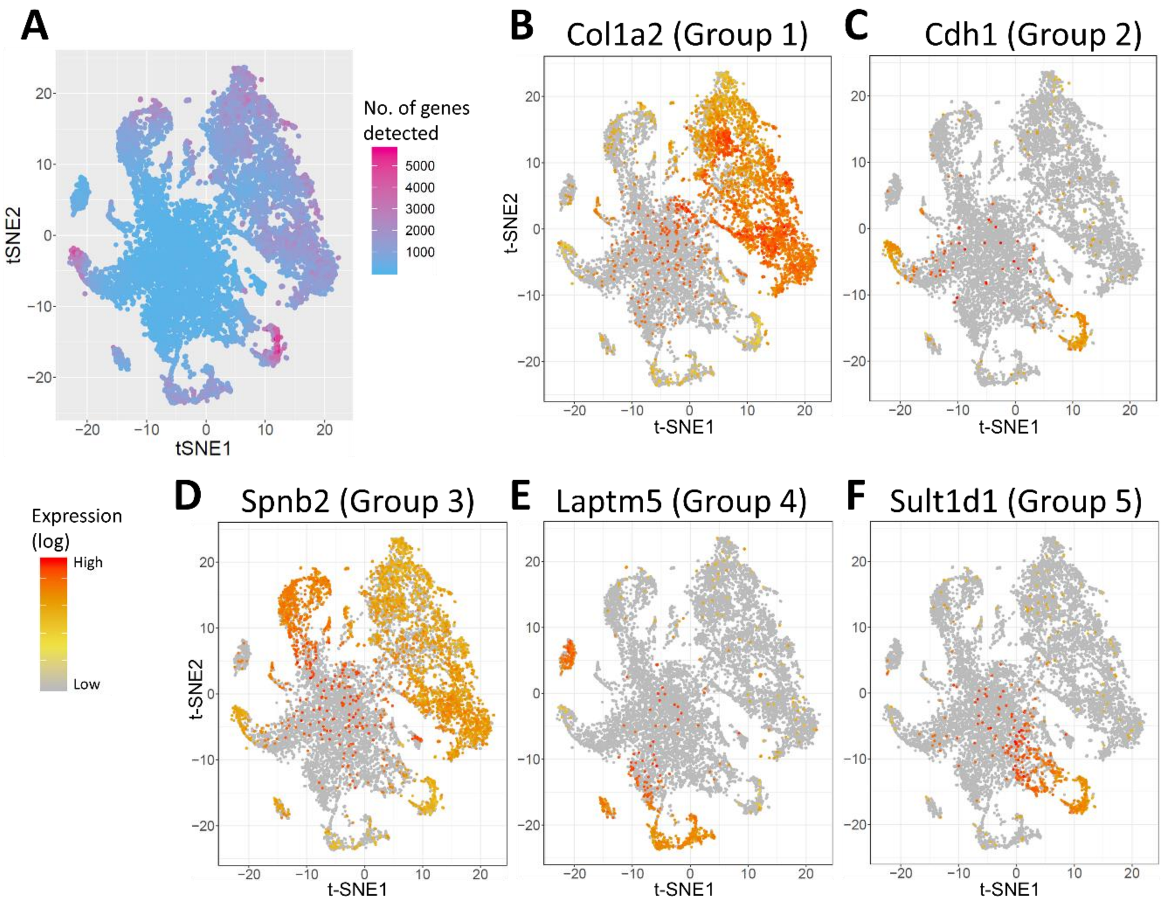

Supplementary Figure 5: Application of singleCellHaystack on Tabula Muris (microfluidic droplet) Trachea (P8_14) tissue dataset. (A) t-SNE plot of the 12,033 cells. The color scale shows the number of genes detected in each cell. **(B-F)** Expression pattern of five top-scoring DEGs, representative of the five groups in which the genes were clustered.

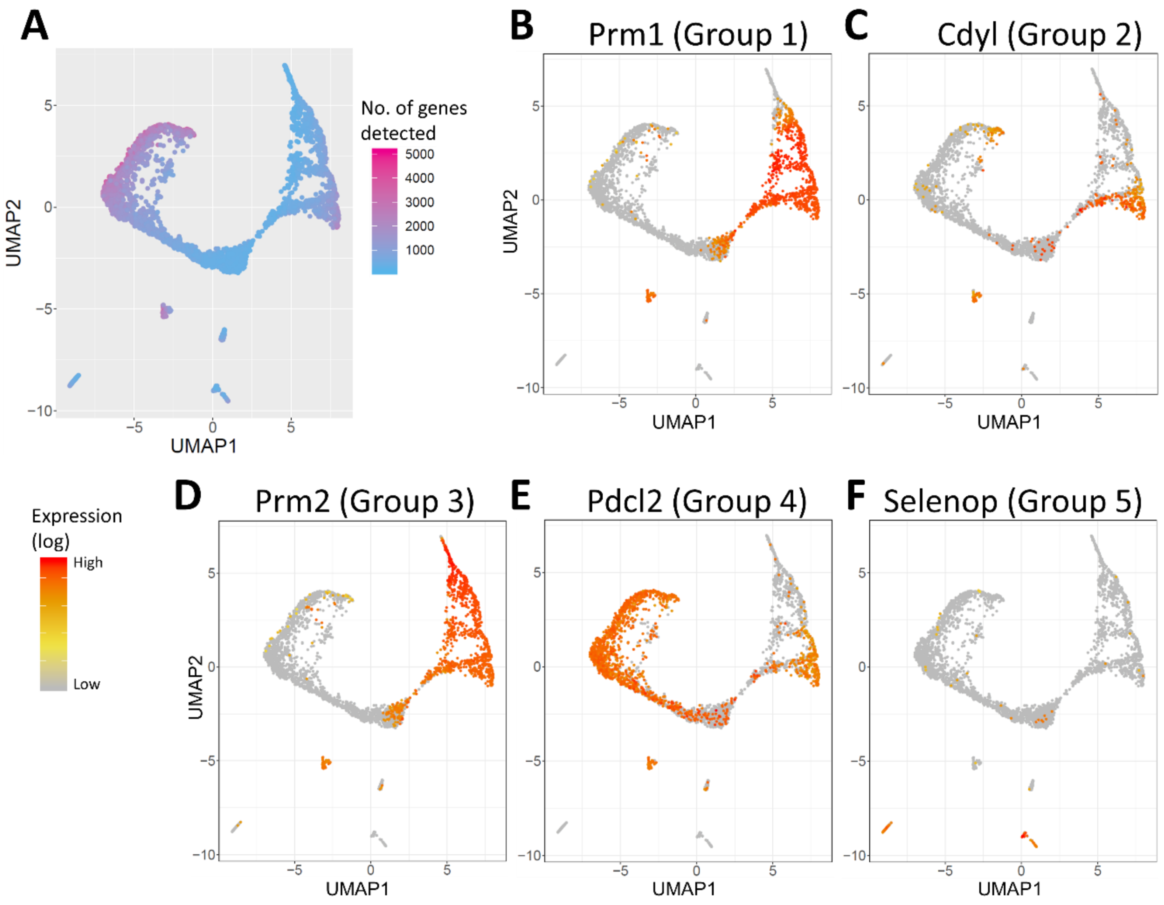

Supplementary Figure 6: Application of singleCellHaystack on Mouse Cell Atlas testis 1 dataset. (A) UMAP plot of the 3,217 cells. The color scale shows the number of genes detected in each cell. **(B-F)** Expression pattern of five top-scoring DEGs, representative of the five groups in which the genes were clustered.

(next page) Supplementary Figure 7: Dependency on parameters. The Tabula Muris bone marrow and trachea datasets and the mouse Cell Atlas testis 1 dataset are used as representative cases. Pairwise scatterplots show the p-values ($\log_{10}$) of all genes and the Spearman correlation between them for runs of singleCellHaystack using **(A)** difference input spaces (t-SNE, UMAP, and the first 5, 10 or 50 PCs), **(B)** different bandwidths (default bandwidth h, "very broad" uses $2 \times h$, "broad" uses $1.5 \times h$, "narrow" uses $h/1.5$, and "very narrow" uses $h/2$), **(C)** different numbers of grid points (25, 50, 100 (default), 150 and 200 grid points), and **(D)** different grid point coordinates (using 5 different values as seed for R's random number generator). **(E)** Similar pairwise scatterplots for the default DEG prediction method (Wilcoxon Rank Sum test) in the Seurat toolkit for different numbers of clusters. Plots show $\log_{10}$(pvalue+1e-300) to avoid log(0) problems. The default number of clusters were 18 for bone marrow, 26 for trachea, and 11 for testis. A color scale is used to indicate large changes in p-values ($\log_{10}$).

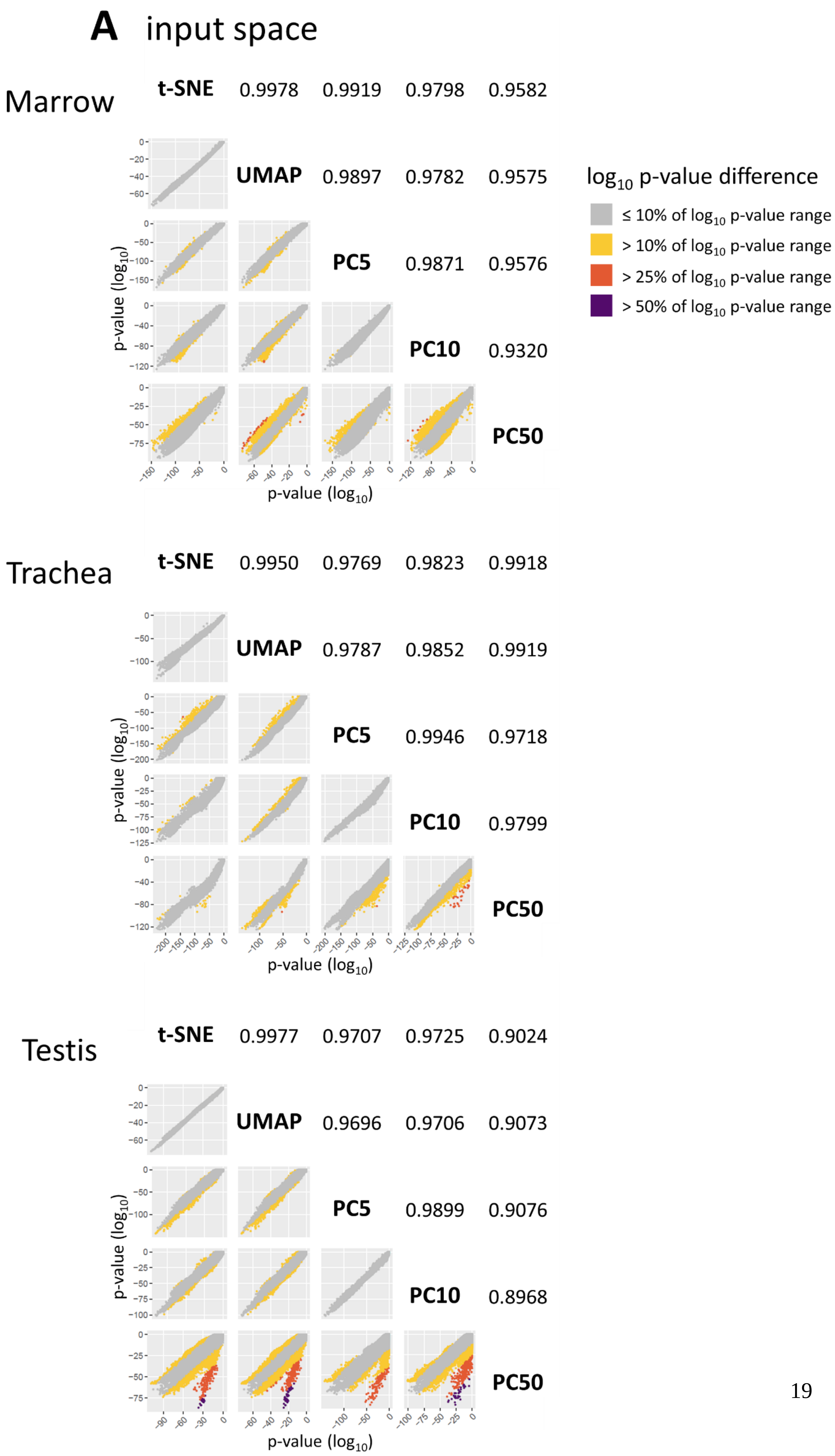
A input space
Marrow
t-SNE 0.9978 0.9919 0.9798 0.9582
UMAP 0.9897 0.9782 0.9575
PC5 0.9871 0.9576
PC10 0.9320
PC50
Trachea
t-SNE 0.9950 0.9769 0.9823 0.9918
UMAP 0.9787 0.9852 0.9919
PC5 0.9946 0.9718
PC10 0.9799
PC50
Testis
t-SNE 0.9977 0.9707 0.9725 0.9024
UMAP 0.9696 0.9706 0.9073
PC5 0.9899 0.9076
PC10 0.8968
PC50
p-value (log_{10})
log10 p-value difference
≤ 10% of log_{10} p-value range
> 10% of log_{10} p-value range
> 25% of log_{10} p-value range
> 50% of log_{10} p-value range

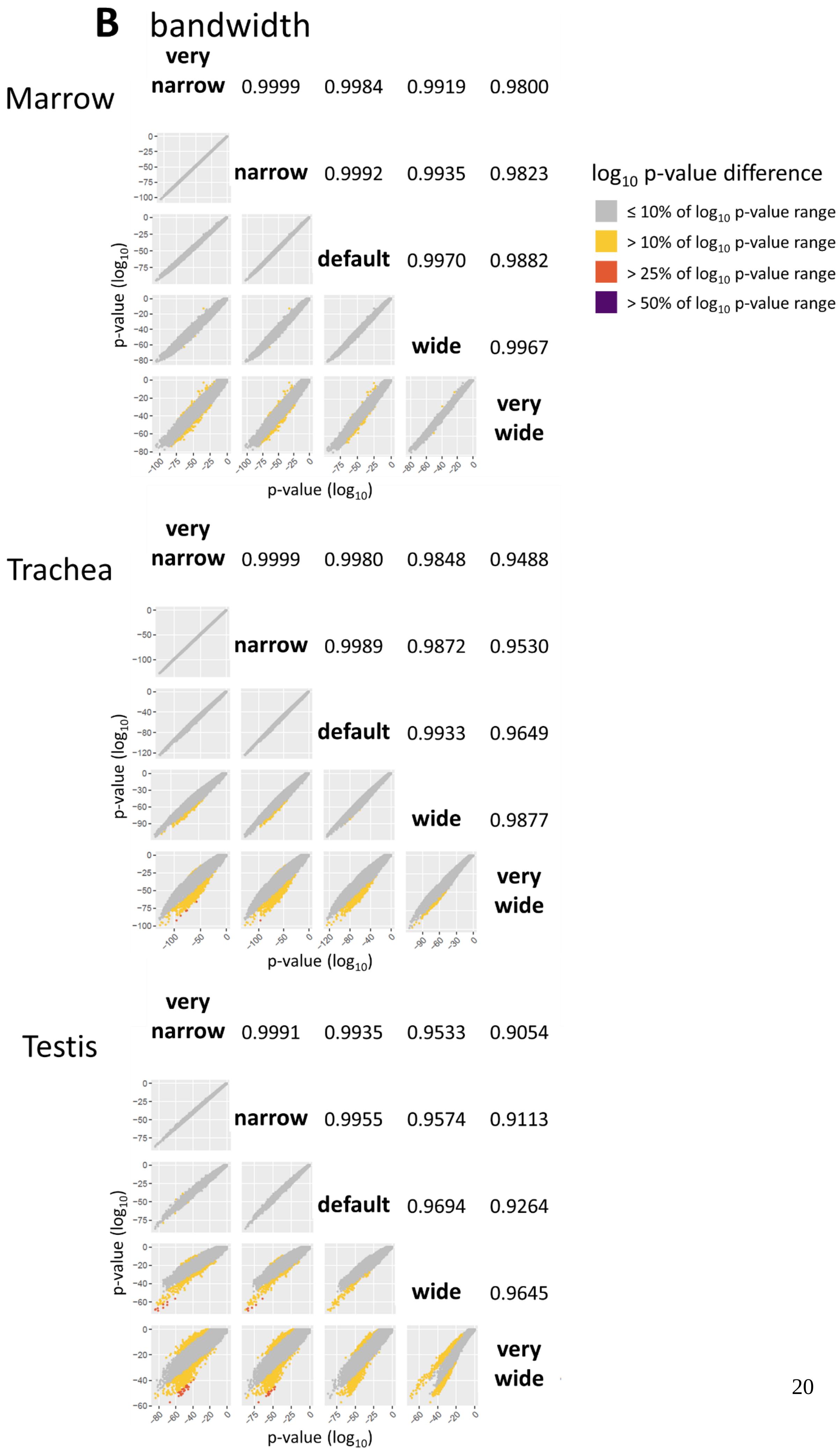
B
bandwidth
Marrow
very narrow 0.9999 0.9984 0.9919 0.9800
narrow 0.9992 0.9935 0.9823
default 0.9970 0.9882
wide 0.9967
very wide
Trachea
very narrow 0.9999 0.9980 0.9848 0.9488
narrow 0.9989 0.9872 0.9530
default 0.9933 0.9649
wide 0.9877
very wide
Testis
very narrow 0.9991 0.9935 0.9533 0.9054
narrow 0.9955 0.9574 0.9113
default 0.9694 0.9264
wide 0.9645
very wide
p-value (log_{10})
log_{10} p-value difference
≤ 10% of log_{10} p-value range
> 10% of log_{10} p-value range
> 25% of log_{10} p-value range
> 50% of log_{10} p-value range

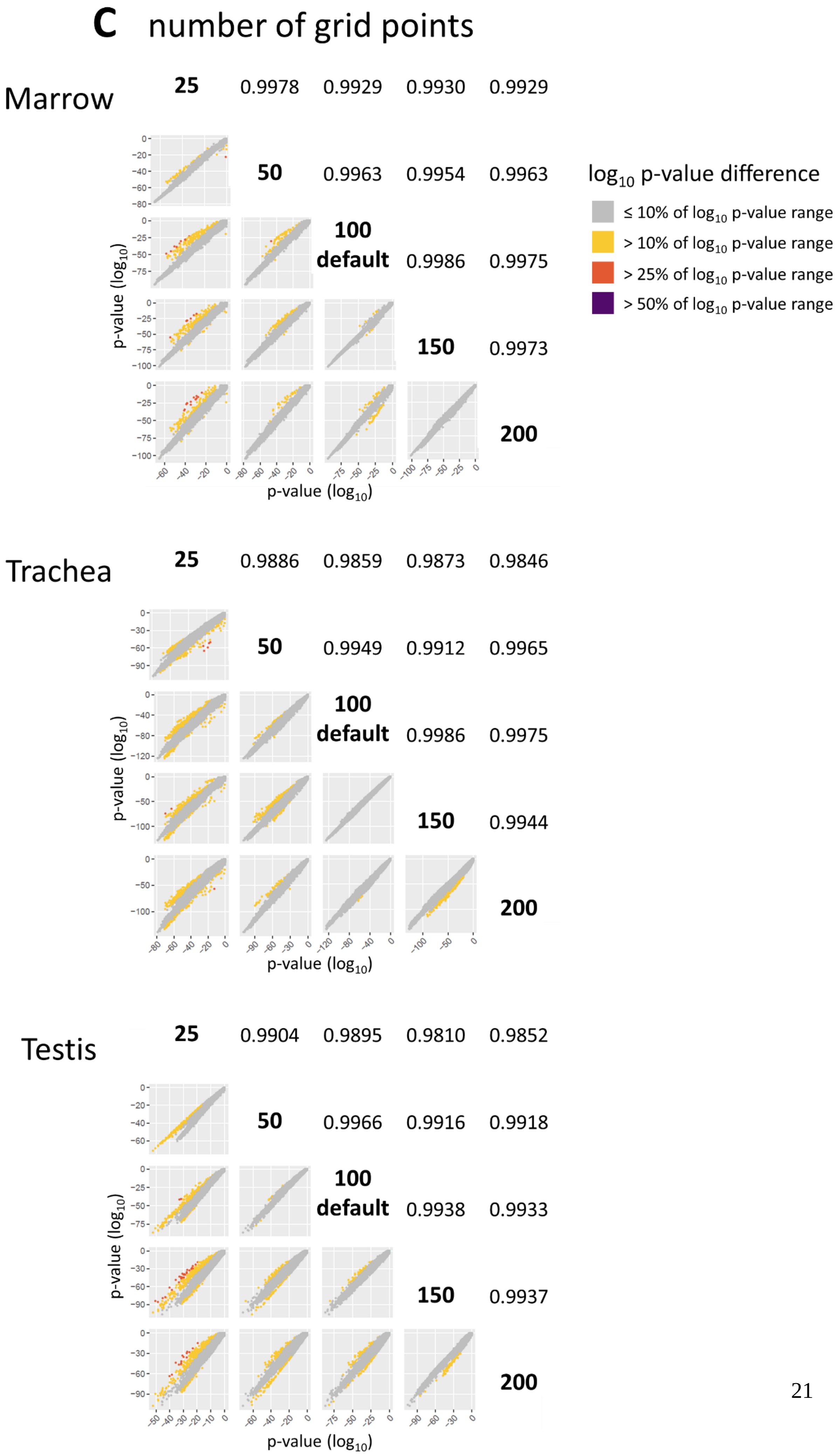
C
number of grid points
Marrow
25
0.9978
0.9929
0.9930
0.9929
50
0.9963
0.9954
0.9963
100
default
0.9986
0.9975
150
0.9973
200
p-value (log10)
p-value (log10)
log10 p-value difference
≤ 10% of log10 p-value range
> 10% of log10 p-value range
> 25% of log10 p-value range
> 50% of log10 p-value range
Trachea
25
0.9886
0.9859
0.9873
0.9846
50
0.9949
0.9912
0.9965
100
default
0.9986
0.9975
150
0.9944
200
p-value (log10)
p-value (log10)
Testis
25
0.9904
0.9895
0.9810
0.9852
50
0.9966
0.9916
0.9918
100
default
0.9938
0.9933
150
0.9937
200
p-value (log10)
p-value (log10)

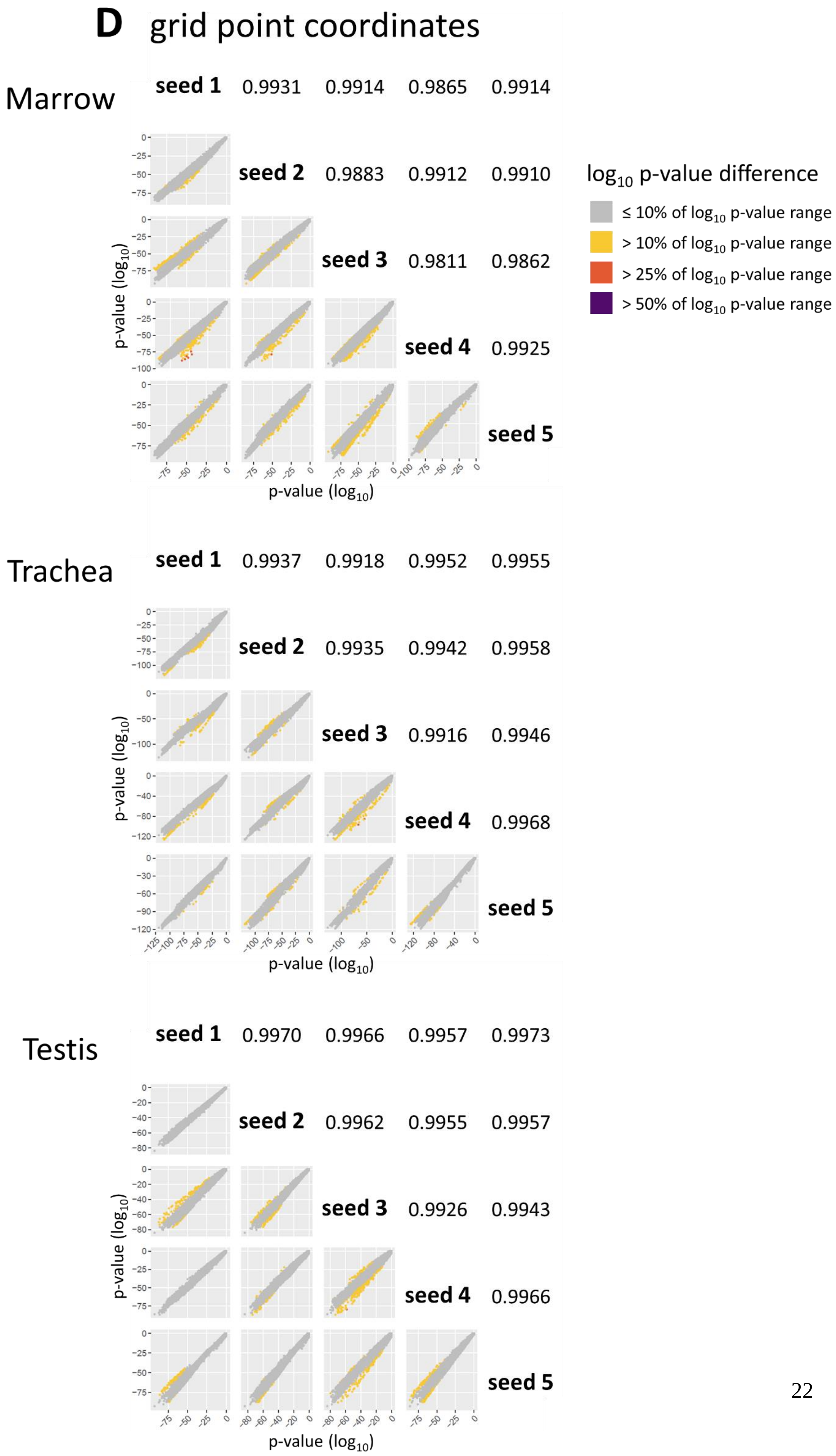
D grid point coordinates
Marrow
seed 1 0.9931 0.9914 0.9865 0.9914
seed 2 0.9883 0.9912 0.9910
seed 3 0.9811 0.9862
seed 4 0.9925
seed 5
p-value (log10)
p-value (log10)
Trachea
seed 1 0.9937 0.9918 0.9952 0.9955
seed 2 0.9935 0.9942 0.9958
seed 3 0.9916 0.9946
seed 4 0.9968
seed 5
p-value (log10)
p-value (log10)
Testis
seed 1 0.9970 0.9966 0.9957 0.9973
seed 2 0.9962 0.9955 0.9957
seed 3 0.9926 0.9943
seed 4 0.9966
seed 5
p-value (log10)
p-value (log10)
log10 p-value difference
≤ 10% of log10 p-value range
> 10% of log10 p-value range
> 25% of log10 p-value range
> 50% of log10 p-value range

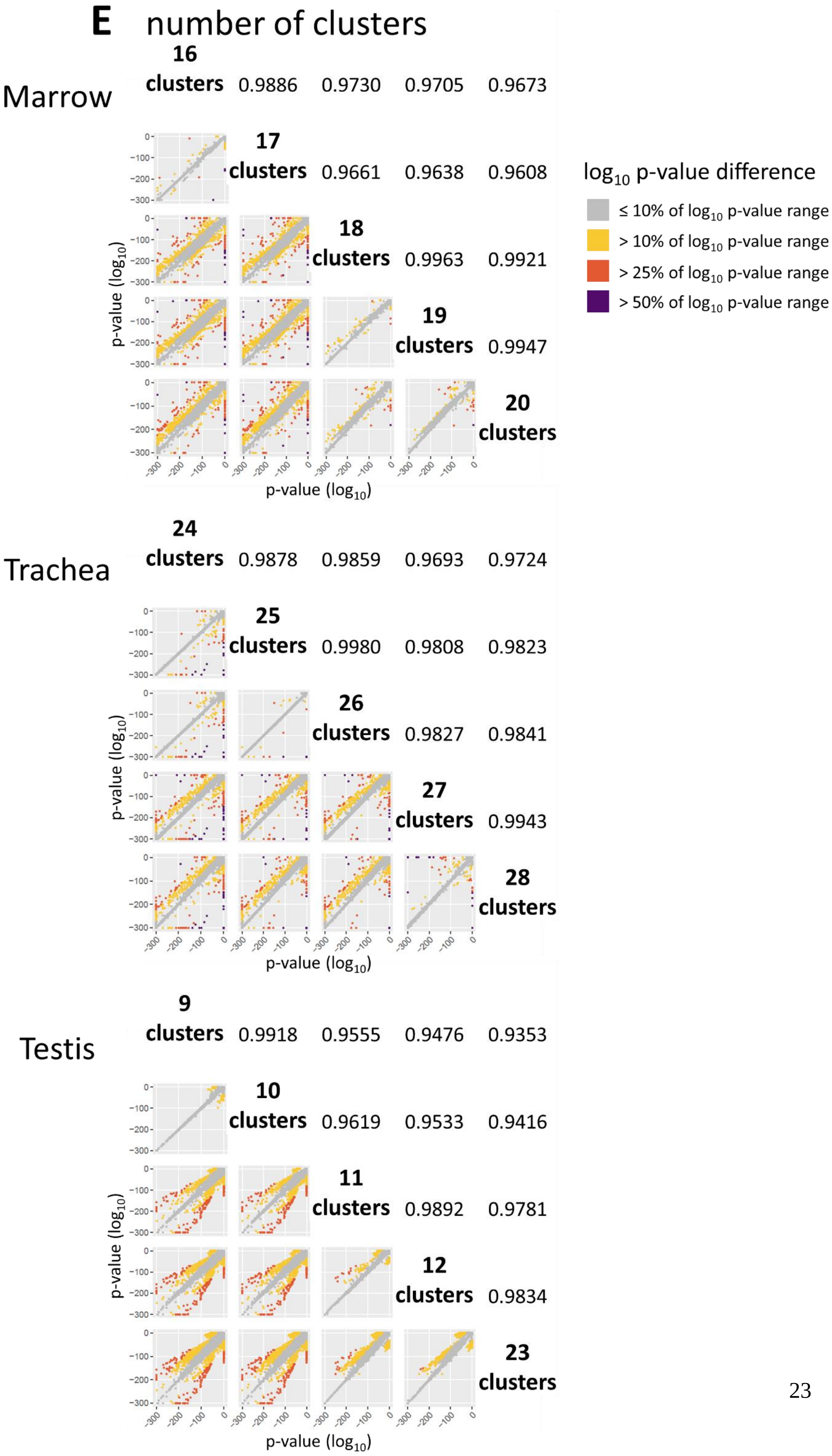
E
number of clusters
Marrow
16 clusters 0.9886 0.9730 0.9705 0.9673
17 clusters 0.9661 0.9638 0.9608
18 clusters 0.9963 0.9921
19 clusters 0.9947
20 clusters
Trachea
24 clusters 0.9878 0.9859 0.9693 0.9724
25 clusters 0.9980 0.9808 0.9823
26 clusters 0.9827 0.9841
27 clusters 0.9943
28 clusters
Testis
9 clusters 0.9918 0.9555 0.9476 0.9353
10 clusters 0.9619 0.9533 0.9416
11 clusters 0.9892 0.9781
12 clusters 0.9834
23 clusters
p-value (log_{10})
log$_{10}$ p-value difference
≤ 10% of log$_{10}$ p-value range
> 10% of log$_{10}$ p-value range
> 25% of log$_{10}$ p-value range
> 50% of log$_{10}$ p-value range

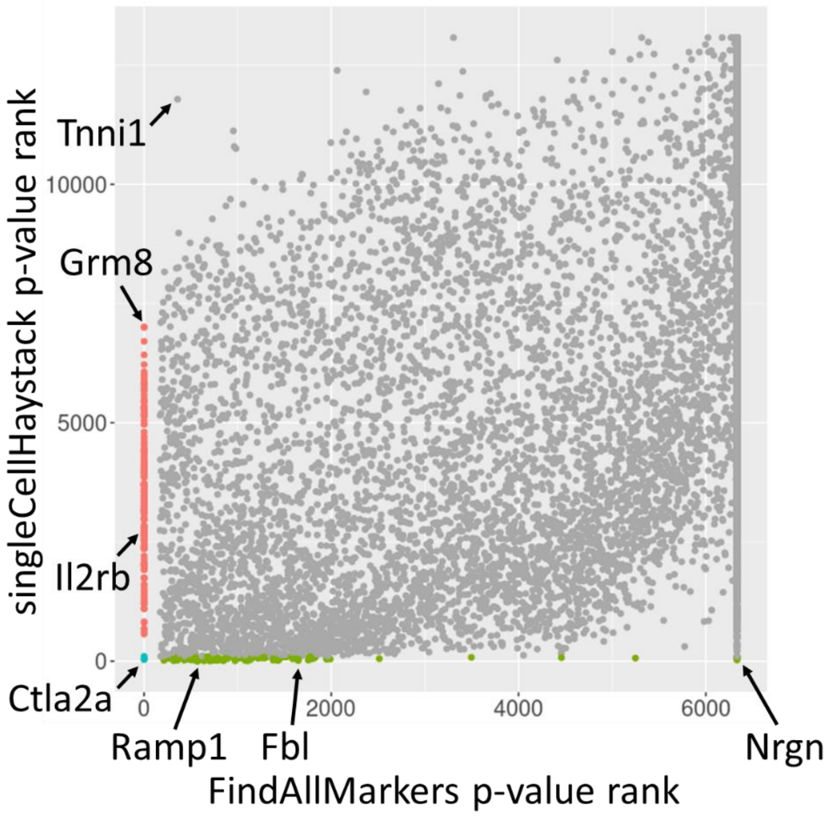

Supplementary Figure 8: Comparison of ranking of genes by singleCellHaystack and Seurat's `FindAllMarkers` function on the Tabula Muris bone marrow tissue dataset. Scatterplot of the ranks of p-values estimated by `FindAllMarkers` (X-axis) and singleCellHaystack (Y-axis) for all 13,756 genes in the dataset. Figure 4A in the main manuscript shows the scatterplot of p-values on which these rankings are based. Red: 176 genes with p-value of 0 by `FindAllMarkers`; Green: top 100 genes with highest significance according to singleCellHaystack. Cyan: three genes in the intersect of the above two sets of genes.

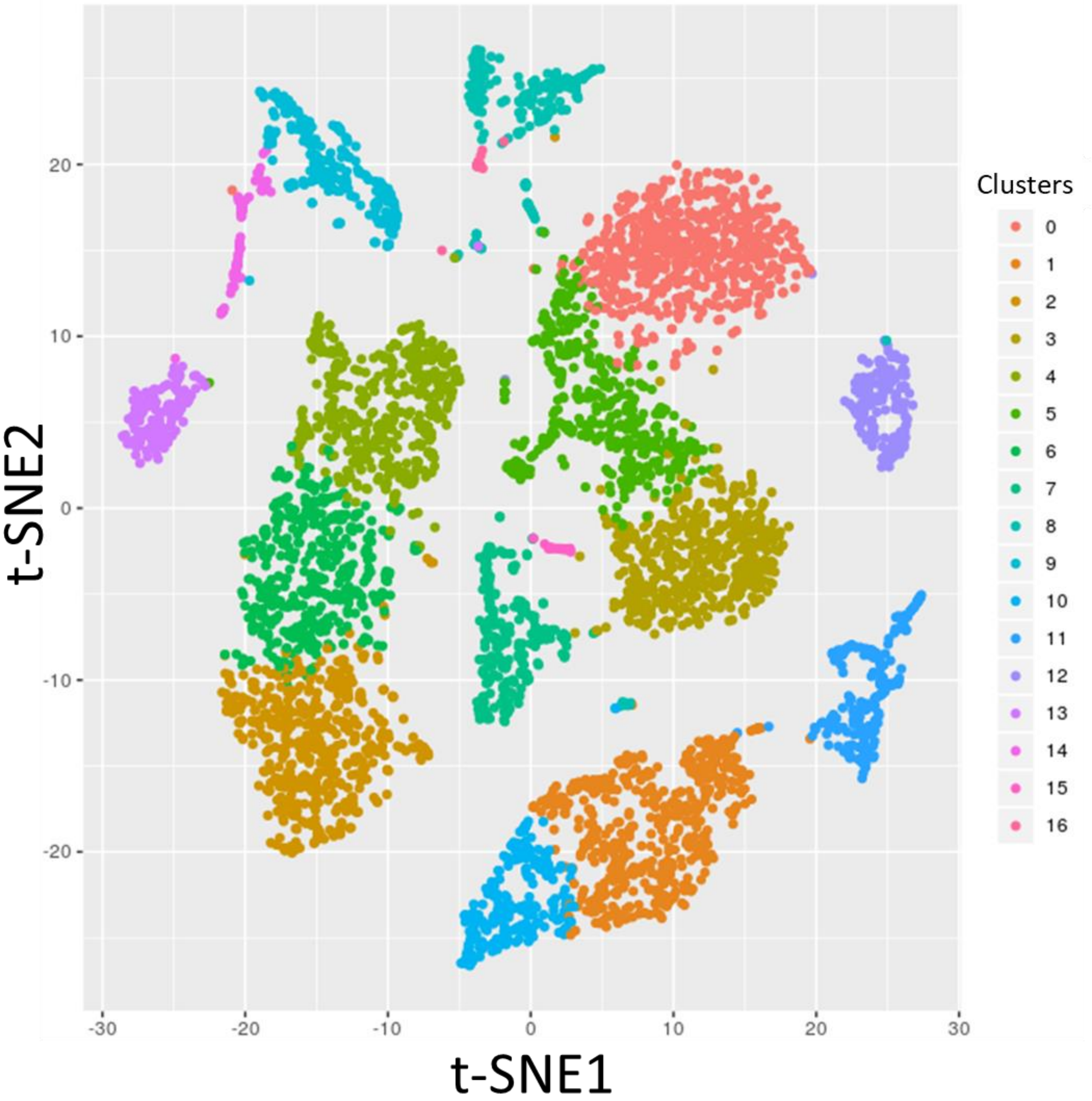

Supplementary Figure 9: Clusters in the Tabula Muris bone marrow dataset decided by Seurat's `FindClusters` function. Different colors represent different clusters of cells. There were 17 clusters predicted in this dataset.

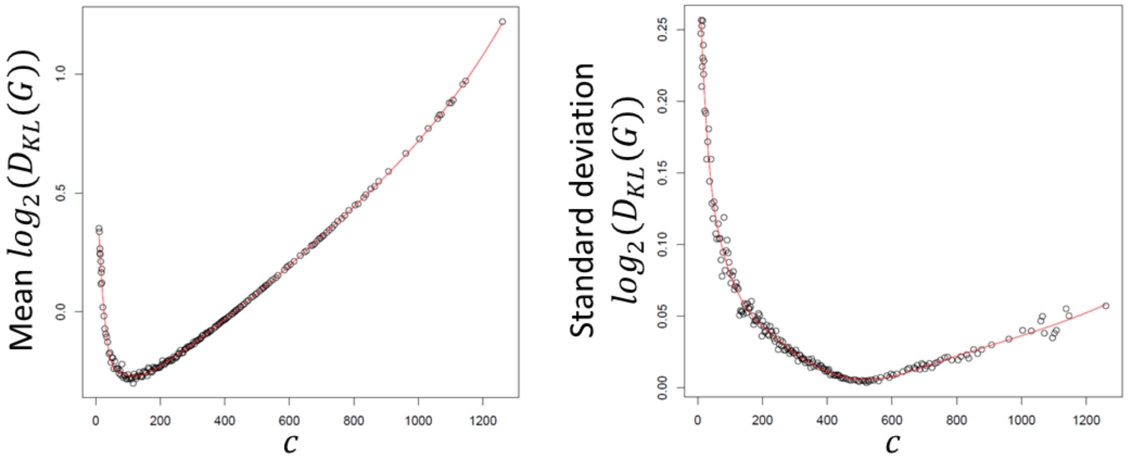

Supplementary Figure 10: Example of trends of the mean and standard deviation of $D_{KL}(G)$. The example shows the mean and standard deviation obtained from randomizations of the Mouse Cell Atlas muscle tissue dataset. Mean and standard deviations are shown for each value of c (the number of cells in which a gene was detected) used in the randomizations. Fitted B-splines are shown in red.

Supplementary References

1. Arthur, D. & Vassilvitskii, S. k-means++ : The Advantages of Careful Seeding. *Proc. eighteenth Annu. ACM-SIAM Symp. Discret. algorithms* 1027–1035 (2007).
2. Kobak, D. & Berens, P. The art of using t-SNE for single-cell transcriptomics. *Nat. Commun.* **10**, 5416 (2019).
3. Schaum, N. *et al.* Single-cell transcriptomics of 20 mouse organs creates a Tabula Muris. *Nature* **562**, 367–372 (2018).
4. Han, X. *et al.* Mapping the Mouse Cell Atlas by Microwell-Seq. *Cell* **172**, 1091–1097 (2018).
5. Nestorowa, S. *et al.* A single-cell resolution map of mouse hematopoietic stem and progenitor cell differentiation. *Blood* **128**, e20–e31 (2016).
6. Krijthe, J. H. Rtsne: T-Distributed Stochastic Neighbor Embedding using a Barnes-Hut Implementation. *https://github.com/jkrijthe/Rtsne* (2015). Available at: https://github.com/jkrijthe/Rtsne.
7. Konopka, T. umap: Uniform Manifold Approximation and Projection. *https://CRAN.R-project.org/package=umap* (2018).
8. Miao, Z., Deng, K., Wang, X. & Zhang, X. DEsingle for detecting three types of differential expression in single-cell RNA-seq data. *Bioinformatics* **34**, 3223–3224 (2018).
9. Nabavi, S., Schmolze, D., Maitituoheti, M., Malladi, S. & Beck, A. H. EMDomics: A robust and powerful method for the identification of genes

differentially expressed between heterogeneous classes. *Bioinformatics* **32**, 533–541 (2016).

10. Korthauer, K. D. *et al.* A statistical approach for identifying differential distributions in single-cell RNA-seq experiments. *Genome Biol.* **17**, 1–15 (2016).

11. McCarthy, D. J., Chen, Y. & Smyth, G. K. Differential expression analysis of multifactor RNA-Seq experiments with respect to biological variation. *Nucleic Acids Res.* **40**, 4288–97 (2012).

12. Qiu, X. *et al.* Single-cell mRNA quantification and differential analysis with Census. *Nat. Methods* **14**, 309–315 (2017).

13. Finak, G. *et al.* MAST: a flexible statistical framework for assessing transcriptional changes and characterizing heterogeneity in single-cell RNA sequencing data. *Genome Biol.* **16**, (2015).

14. Butler, A., Hoffman, P., Smibert, P., Papalexi, E. & Satija, R. Integrating single-cell transcriptomic data across different conditions, technologies, and species. *Nat. Biotechnol.* **36**, 411–420 (2018).

15. McDavid, A. *et al.* Data exploration, quality control and testing in single-cell qPCR-based gene expression experiments. *Bioinformatics* **29**, 461–467 (2013).
