# Supplementary material for: A clustering-independent method for finding differentially expressed genes in single-cell transcriptome data
Source: Nat Commun. 2020 Aug 28;11:4318. doi: 10.1038/s41467-020-17900-3 (PMC7455704; doi:10.1038/s41467-020-17900-3)
Supplement: Supplementary file 3 — Reporting Summary [file 41467_2020_17900_MOESM3_ESM.pdf]

nature research

Corresponding author(s): Alexis Vandenbon

Last updated by author(s): Jul 20, 2020

# Reporting Summary

Nature Research wishes to improve the reproducibility of the work that we publish. This form provides structure for consistency and transparency in reporting. For further information on Nature Research policies, see Authors & Referees and the Editorial Policy Checklist .

## Statistics

For all statistical analyses, confirm that the following items are present in the figure legend, table legend, main text, or Methods section.

| n/a | Confirmed | |
|---|---|---|
| ☒ | ☐ | The exact sample size (*n*) for each experimental group/condition, given as a discrete number and unit of measurement |
| ☒ | ☐ | A statement on whether measurements were taken from distinct samples or whether the same sample was measured repeatedly |
| ☒ | ☐ | The statistical test(s) used AND whether they are one- or two-sided<br>*Only common tests should be described solely by name; describe more complex techniques in the Methods section.* |
| ☒ | ☐ | A description of all covariates tested |
| ☐ | ☒ | A description of any assumptions or corrections, such as tests of normality and adjustment for multiple comparisons |
| ☒ | ☐ | A full description of the statistical parameters including central tendency (e.g. means) or other basic estimates (e.g. regression coefficient) AND variation (e.g. standard deviation) or associated estimates of uncertainty (e.g. confidence intervals) |
| ☒ | ☐ | For null hypothesis testing, the test statistic (e.g. *F*, *t*, *r*) with confidence intervals, effect sizes, degrees of freedom and *P* value noted<br>*Give P values as exact values whenever suitable.* |
| ☒ | ☐ | For Bayesian analysis, information on the choice of priors and Markov chain Monte Carlo settings |
| ☒ | ☐ | For hierarchical and complex designs, identification of the appropriate level for tests and full reporting of outcomes |
| ☒ | ☐ | Estimates of effect sizes (e.g. Cohen's *d*, Pearson's *r*), indicating how they were calculated |

*Our web collection on statistics for biologists contains articles on many of the points above.*

## Software and code

Policy information about availability of computer code

| | |
|---|---|
| Data collection | No software was used for data collection. |
| Data analysis | Data was analyzed using R (version 3.6.0) and RStudio (1.1.383). The method described in the paper is available as an R package on GitHub (https://github.com/alexisvdb/singleCellHaystack) and on CRAN (https://CRAN.R-project.org/package=singleCellHaystack) under an MIT License. |

For manuscripts utilizing custom algorithms or software that are central to the research but not yet described in published literature, software must be made available to editors/reviewers. We strongly encourage code deposition in a community repository (e.g. GitHub). See the Nature Research guidelines for submitting code & software for further information.

## Data

Policy information about availability of data

All manuscripts must include a data availability statement. This statement should provide the following information, where applicable:

- Accession codes, unique identifiers, or web links for publicly available datasets
- A list of figures that have associated raw data
- A description of any restrictions on data availability

The artificial single-cell datasets generated using Splatter for the comparison of singleCellHaystack with other DEG prediction methods are available in figshare with the identifiers 10.6084/m9.figshare.12319787 and 10.6084/m9.figshare.12319247. Other single-cell RNA-seq datasets analyzed in this study are available from https://tabula-muris.ds.czbiohub.org/ (Tabula Muris), https://figshare.com/articles/MCA_DGE_Data/5435866 (Mouse Cell Atlas), and GEO accession number GSE81682. The cell type marker data was obtained from the CellMarker database and is available from http://biocc.hrbmu.edu.cn/CellMarker/.

# Field-specific reporting

Please select the one below that is the best fit for your research. If you are not sure, read the appropriate sections before making your selection.

- [x] Life sciences
- [ ] Behavioural & social sciences
- [ ] Ecological, evolutionary & environmental sciences

For a reference copy of the document with all sections, see nature.com/documents/nr-reporting-summary-flat.pdf

# Life sciences study design

All studies must disclose on these points even when the disclosure is negative.

| | |
|---|---|
| Sample size | A collection of 136 single-cell datasets from previously published studies were used. No changes to the size (numbers of cells or numbers of genes) of datasets were made, except for datasets with more than 20,000 cells. For those datasets, we selected 20,000 cells for analysis (as described in Supplementary material). We did this because we wanted to focus on dataset sizes that are representative of typical current single-cell datasets. Sizes of artificial datasets were chosen to be similar to real datasets. |
| Data exclusions | No data was excluded. For the analysis of single-cell data, a typical processing workflow was used which does include filtering out cells with few detected genes and genes detected in few cells. |
| Replication | Our analysis was performed on published datasets. We did not attempt to replicate published studies. However, to show the consistency of our method, we applied our method using 5 different random seeds, and different input parameters (5 different input spaces, 5 different values of the bandwidth parameter, 5 different numbers of grid points), and compared the results. Details are described in the paper, and show that our method in general returns consistent results. |
| Randomization | This is not relevant to our study. Our study does not involve experimental groups and comparisons between them. |
| Blinding | This is not relevant to our study. Our study does not involve experimental groups and comparisons between them which would require blinding. |

# Reporting for specific materials, systems and methods

We require information from authors about some types of materials, experimental systems and methods used in many studies. Here, indicate whether each material, system or method listed is relevant to your study. If you are not sure if a list item applies to your research, read the appropriate section before selecting a response.

## Materials & experimental systems

| n/a | Involved in the study |
|---|---|
| ☒ | ☐ Antibodies |
| ☒ | ☐ Eukaryotic cell lines |
| ☒ | ☐ Palaeontology |
| ☒ | ☐ Animals and other organisms |
| ☒ | ☐ Human research participants |
| ☒ | ☐ Clinical data |

## Methods

| n/a | Involved in the study |
|---|---|
| ☒ | ☐ ChIP-seq |
| ☒ | ☐ Flow cytometry |
| ☒ | ☐ MRI-based neuroimaging |
